# Meningoencephalitis: A Rare Presentation of Scrub Typhus

**DOI:** 10.7759/cureus.29597

**Published:** 2022-09-26

**Authors:** Ayushma Acharya, Tulsiram Bhattarai

**Affiliations:** 1 Emergency Medicine, Helping Hands Community Hospital, Kathmandu, NPL; 2 Medicine, Kathmandu Medical College and Teaching Hospital, Kathmandu, NPL

**Keywords:** nepal, doxycycline, febrile illness, scrub typhus, meningoencephalitis

## Abstract

Scrub typhus is a mite-born acute febrile illness endemic to a part of the world known as the “tsutsugamushi triangle,” which also includes Nepal. It has a wide range of presentations with multiple organ involvement, including meningoencephalitis. We present a unique case of a 30-year-old lady with scrub typhus meningoencephalitis, which showed drastic improvement with doxycycline. This case highlights the importance of high suspicion in an endemic area with limited diagnostic facilities. With the early initiation of empirical therapy, fatal complications of scrub typhus infection such as meningoencephalitis can be prevented.

## Introduction

Scrub typhus, also known as tsutsugamushi disease, is a mite-born acute febrile illness caused by *Orientia tsutsugamushi*. It is usually characterized by fever, myalgia, rash, headache, hepatosplenomegaly, and lymphadenopathy. It has multisystem involvement, including the central nervous system, leading to meningoencephalitis [1]. Clinical features differentiating meningitis secondary to scrub typhus from other forms of acute bacterial and viral meningitis are not very clear [2]. The pathognomonic eschar, indicative of the vector bite, is present in less than half of the patients with scrub typhus. Some other manifestations include acute respiratory distress syndrome, myocarditis, acute kidney injury, hepatitis, and lymphadenopathy [3].

## Case presentation

A 30-year-old lady with a history of seizure disorder for five years with medication noncompliance presented to our emergency department as a referral from a rural hospital with a witnessed episode of a generalized tonic-clonic seizure. The patient initially complained of high-grade intermittent fever with chills for one week and a severe constant headache with an altered mental state for two days. She had multiple episodes of nonbloody, nonbilious projectile vomiting. She was put on vancomycin and piperacillin at the previous hospital. On our initial evaluation, she was awake but not oriented to time, place, and person. She was following commands poorly, and the Glasglow Coma Scale score was 13 (eye - 3, motor - 4, verbal - 6). She was afebrile, her heart rate was 90 beats per minute, her blood pressure was 130/80 mmHg, her respiratory rate was 18 breaths per minute, and her saturation was 96% on room air. Signs of meningeal irritation were present. Lung auscultation revealed bilateral infra-axillary rales. Cardiovascular and abdominal examinations were within normal limits.

As shown in Table 1, the initial laboratory workup revealed anemia, thrombocytopenia, elevated transaminase, and elevated total bilirubin. Acute hepatitis panel and human immunodeficiency virus serology were negative. Coronavirus disease 2019 nasopharyngeal swab was negative. Chest X-ray showed patchy infiltrates in the right lower lobe of the lung, suggestive of pneumonia. Sputum cytology was sent, and the patient was started on meropenem and vancomycin. Computed tomography of the head was negative for any stroke. Electroencephalogram showed generalized encephalopathy with the interictal pattern. Lumbar puncture was performed with the ongoing altered mental status. Cerebrospinal fluid (CSF) analysis showed no organism on gram stain, potassium hydroxide preparation, and culture. However, CSF fluid adenosine deaminase (ADA) was elevated. Real-time polymerase chain reaction (PCR) of CSF fluid and sputum GeneXpert for tuberculosis were negative.

**Table 1:** Investigation findings on admission.

Parameter	Results	Reference range
White blood cell count	9.7 × 10^3^/µL	4–11 × 10^3^/µL
Hemoglobin	10.9 g/dL	11.8–14.8 g/dL
Platelets	110,000/mm^3^	150,000–400,000/mm^3^
Alkaline phosphatase	778 IU/L	30–120 IU/L
Alanine aminotransferase	374 IU/L	0–35 IU/L
Aspartate transaminase	334 IU/L	8–33 IU/L
Total bilirubin	2.0 mg/dL	0.3–1.2 mg/dL
Blood culture	No organism growth	NA
Random blood sugar	110 mmol/L	<140 mmol/L

On day four, the patient had a persistent headache, fever, transaminase, and worsening thrombocytopenia. The blood culture did not grow any organism. However, sputum culture isolated *Klebsiella pneumoniae*. With persistent febrile illness and transaminitis, extensive workup was started for febrile illness but was negative for malaria, dengue, *Brucella*, *Leptospira*, and *Salmonella*. However, immunoglobulin M (IgM) and immunoglobulin G (IgG) antibodies for scrub typhus were positive. Doxycycline was started, following which the patient became afebrile at 24 hours. Transaminitis and thrombocytopenia improved over a few days, and the patient was discharged on the 17th day of admission. Hence, the patient’s presentation of sepsis with multisystem involvement, including meningoencephalitis, was due to a scrub typhus infection.

## Discussion

Scrub typhus is an underreported mite-born zoonotic disease, which is a usually underdiagnosed cause of acute febrile illness. It has a variable presentation with multiorgan involvement. The multiorgan involvement in scrub typhus is due to the relative propensity of *O. tsutsugamushi* to infect vascular endothelial cells, which results in microinfarct formation [4]. Direct invasion of the central nervous system (CNS) has been noted [5]. CNS involvement has been noted in almost all rickettsial diseases, of which *O. tsutsugamushi* and *Coxiella Brunetti* predominately manifest as meningoencephalitis [3,4].

The disease has many nonspecific features that clinicians misdiagnose with enteric fever, *Leptospira*, or tuberculosis. Elevated liver enzymes are common findings associated with scrub typhus and help point toward diagnosis [3,6,7]. Although the eschar is the pathognomic lesion found in 20-80% of cases, our patient did not have it. This can be because the identification of eschar is difficult in South Asians due to their dark skin, and it may be inconspicuous as it is often present in areas such as the groin, gluteal folds, breast folds, and external genitalia. It is also expected to be less in areas endemic to the disease. Nepal falls under the “tsutsugamushi triangle,” which extends from Pakistan, India, and Nepal in the west to South-Eastern Siberia, Japan, China, and Korea in the north to Indonesia, the Philippines, Northern Australia, and the Pacific Islands in the south which are endemic to the disease [3,6].

The presence of fever for more than five days with CSF pleocytosis, CSF lymphocytosis >50% of cases, and an increase in Alanine aminotransferase (ALT) of more than 60 IU are suggestive lab parameters for scrub typhus meningitis. Our patient did have a fever and elevated ALT, but her CSF was normal with no pleocytosis. However, CSF ADA was elevated. This CSF picture can be due to partially treated meningitis but can also occur in tuberculous meningitis. Although markers such as elevated CSF ADA are associated with tubercular meningitis, there are incidences of elevated CSF ADA in cases of scrub typhus meningitis. Hence, another test for ruling out tuberculosis is necessary as the two can often be confusing [3,4,8]. Real-time PCR of CSF and sputum GeneXpert for mycobacteria was negative in the patient.

Hence, the diagnosis is mainly based on high clinical suspicion in an endemic area with suggestive clinical features and positive serological tests for scrub typhus. Although the organism can be detected in clinical specimens, serological tests are indispensable for diagnosis in developing countries [9]. Our patient presented with severe headaches and seizures, which have been used as vital clinical criteria to identify suspected cases [4,8]. Serological tests for scrub typhus that detected IgM and IgG antibodies and a dramatic improvement in symptoms following treatment with doxycycline helped diagnose our case.

Comparing our case with recent literature, we found that our patient had a triad of fever, altered mental status, and meningeal signs, consistent with a study conducted by Eswaradass et al. [10]. Based on several previous clinical studies, patients with scrub typhus show early lymphocytosis and thrombocytopenia [10]. Though lymphocytosis was not seen in our patient, thrombocytopenia was present.

## Conclusions

Scrub typhus is underdiagnosed due to its nonspecific clinical presentation, limited awareness, low index of suspicion among clinicians, and lack of diagnostic facilities. Hence, such rare differential diagnoses must be considered when patients do not respond to conventional medical therapy and are from an endemic area. Early diagnosis and administration of appropriate antibiotics can significantly improve the outcomes of the disease.
